# A training programme facilitating guideline use of occupational health professionals: a feasibility study

**DOI:** 10.1186/s12909-018-1223-1

**Published:** 2018-10-03

**Authors:** Marloes Vooijs, Daniël Bossen, Jan L. Hoving, Haije Wind, Monique H. W. Frings-Dresen

**Affiliations:** Amsterdam UMC, University of Amsterdam, Coronel Institute of Occupational Health, Amsterdam Public Health research institute, Amsterdam, The Netherlands

**Keywords:** Occupational health, Occupational medicine, Guideline adherence, Occupational health physicians, Training programme, Medical education, Constructive alignment, Employment

## Abstract

**Background:**

To evaluate whether a training programme is a feasible approach to facilitate occupational health professionals’ (OHPs) use of knowledge and skills provided by a guideline.

**Methods:**

Feasibility was evaluated by researching three aspects: ‘acceptability’, ‘implementation’ and ‘limited efficacy’. Statements on acceptability and implementation were rated by OHPs on 10-point visual analogue scales after following the training programme (T2). Answers were analysed using descriptive statistics. Barriers to and facilitators of implementation were explored through open-ended questions at T2, which were qualitatively analysed. Limited efficacy was evaluated by measuring the level of knowledge and skills at baseline (T0), after reading the guideline (T1) and directly after completing the training programme (T2). Increase in knowledge and skills was analysed using a non-paramatric Friedman test and post-hoc Wilcoxon signed rank tests (two-tailed).

**Results:**

The 38 OHPs found the training programme acceptable, judging that it was relevant (M: 8, SD: 1), increased their capability (M: 7, SD: 1), adhered to their daily practice (M: 8, SD: 1) and enhanced their guidance and assessment of people with a chronic disease (M: 8, SD: 1). OHPs found that it was feasible to implement the programme on a larger scale (M: 7, SD: 1) but foresaw barriers such as ‘time’, ‘money’ and organizational constraints. The reported facilitators were primarily related to the added value of the knowledge and skills to the OHPs’ guidance and assessment, and that the programme taught them to apply the evidence in practice. Regarding limited efficacy, a significant increase was seen in OHPs’ knowledge and skills over time (X^2^ (2) = 53.656, *p* < 0.001), with the median score improving from 6.3 (T0), 8.3 (T1) and 12.3 (T2). Post-hoc tests indicated a significant improvement between T0 and T1 (p < 0.001) and between T1 and T2 (p < 0.001).

**Conclusions:**

The training programme was found to be a feasible approach to facilitate OHPs’ use of knowledge and skills provided by the guideline, from the perspective of OHPs generally (acceptability and implementation) and with respect to their increase in knowledge and skills in particular (limited efficacy).

**Electronic supplementary material:**

The online version of this article (10.1186/s12909-018-1223-1) contains supplementary material, which is available to authorized users.

## Background

Previous research has shown that having a chronic disease negatively affects work participation, as people with a chronic disease are less often employed [1, 2] and, when they are employed, experience difficulties in meeting physical or psychosocial work demands [3]. Occupational health professionals (OHPs) may support such people to improve their work participation. In the Netherlands, there are two types of OHPs involved: occupational physicians (OPs), who provide guidance to individuals to support work retention or return to work, and insurance physicians (IPs), who conduct a work ability assessment of individuals with a chronic disease.

The provision of recent and relevant evidence can support OHPs in their guidance or assessment tasks. Several guidelines have been developed, incorporating recent evidence, with the aim of improving the quality of guidance or assessment given by OHPs [4, 5]. One of these guidelines is the ‘Work participation of people with a chronic disease’ guideline [6], which aims to support the work participation of people with a chronic disease. The guideline includes an overview of factors, interventions and input on collaboration among professionals to promote the work participation of individuals with a chronic disease, irrespective of their specific diagnosis.

Although the use of knowledge and skills provided by a guideline [6] can lead to a higher quality of occupational care [4, 5], guideline adherence by OHPs is generally low [7–9]. Previous studies have shown that guideline use is influenced by various factors that may act as barriers, which are related to the professional, the individual with a chronic disease, or to the knowledge included in the guideline [10, 11]. One of these barriers is a lack of knowledge or skills of OHPs [10, 11], which influences their capability, motivation and opportunity to use the evidence from the guideline in practice [12].

The knowledge and skills provided by a guideline might thus act to enhance practice, but studies recognize that active strategies are needed to increase their uptake and use [13, 14]. In this respect, multiple educational methods have been found to be effective in facilitating learning [14, 15]. On this basis, we developed a training programme to facilitate OHPs’ capability, to increase use of the guideline mentioned above and the knowledge and skills it provided.

Before focusing on implementation on a large scale, Grol and Wensing [16] recommend first testing and running such a training programme with a smaller sample to evaluate whether the programme is a feasible approach to facilitate OHPs’ knowledge and skills. In addition, performing a feasibility study provides valuable information on how the trainees perceive the programme, and whether they consider it to have contributed to their knowledge and daily practice [16, 17].

Bowen [17] states that there are eight aspects which can be addressed in a feasibility study, namely: acceptability, demand, implementation, practicality, adaptation, integration, expansion and limited-efficacy testing [17]. These aspects measure how a training programme is perceived by the trainees, whether the training programme can be carried out as intended, whether it fits with the current system, whether it can be adapted for another target group, and whether it shows promise of being successful. As our aim was to study whether the training programme is feasible in facilitating OHPs’ use of the knowledge and skills provided by the guideline, we focused on the aspects of ‘acceptability’, ‘implementation’ and ‘limited efficacy’.

Acceptability is a common area of interest in feasibility studies [17], which focuses on whether trainees – in our case OHPs – perceive the training programme as helpful and as valuable to their daily practice. We also evaluated the aspect of ‘implementation’ to explore whether trainees perceive that the training programme could be implemented on larger scale. Finally, we studied limited efficacy to evaluate whether, in a smaller sample of the intended population (i.e. OHPs), the training programme shows effectiveness in terms of an improvement in the participants’ knowledge and skills [17]. The study aims to answer the research question: What levels of perceived acceptability, implementation potential and limited efficacy does our training programme for OHPs have, with respect to its aim of facilitating the use of knowledge and skills provided by a guideline?

## Methods

Feasibility of the training programme was evaluated using an observational design. Acceptability and implementation of the training programme were explored after the training programme, as trainees’ perception of the training could only be reported after experiencing the programme. Limited efficacy was measured using a one-group pre-post design by researching the level of knowledge and skills of trainees at baseline (T0), after reading the guideline (T1) and directly after completing the training activities (T2). The Medical Ethics Committee of the Academic Medical Center determined through a written statement that no ethical approval was required for this study (trial number: W17_081#17.100).

### Participants

Based on Bowen et al. [17] and Ruitenburg et al. [18] we aimed to recruit a total of 20–40 participants, to be divided into two training groups at different training locations. As we aimed to include an equal number of OPs and IPs for each training programme, we used stratified sampling. OPs and IPs were recruited by contacting several professionals in the field, including a staff member from the professional association of OPs, a staff member of the national training institute for OHPs, and two staff IPs working in the regions in which the training programme was held. These people then invited OHPs from their network to join the study by sending them an email, including a standardized information letter, which contained all the relevant information about the study, the content of the study and the nature of the training programme. In addition, it stated that participation in the study was voluntary. The OHPs who were interested in participating could register by sending an email to the first researcher (MV). OPs and IPs were included if they had experience in the guidance or assessment of people with a chronic disease. Written informed consent was obtained from all participants included in this study.

### Training programme

The training programme was developed in collaboration with OPs, IPs and experts in the field of education of professionals. The process of the development of the training programme has been reported in another article. In brief, as a first step, OP and IP training needs were explored by asking the OHPs what they would need to use the knowledge and skills provided by the guideline in practice. Based on the OHPs’ reported training needs, researchers formulated learning objectives as a second step (see Table 1). Subsequently, experts in the field of education were interviewed to determine which training activities could be employed to best impart the knowledge and skills to OHPs. Finally, based on the input of both the OHPs and the experts, the learning objectives and teaching methods were integrated into a one-day training programme by the researchers. The training programme was provided by two trainers, an OP and an IP. The first researcher (MV) was present during both training programmes and provided an explanation regarding the content of the guideline as well as assisting the trainers when needed. The second researcher (DB) was present at one training location and assisted the trainers when needed. The protocol of the training programme is presented in Table 1.

**Table 1:** Formulated training programme

Learning objectives			
• OPs/IPs have knowledge of factors influencing work participation • OPs/IPs have knowledge of effective interventions to reduce effect of factors negatively influencing work participation • OPs/IPs evaluate the use of a multi-component intervention at an early stage • OPs/IPs are able to increase the role of the individual through counselling and guidance • OPs/IPs are able to communicate with the employer about the reintegration plan and provide advice on the importance of social support from the workplace • OPs/IPs are able to collaborate together in the guidance and assessment of people with a chronic disease			
Part	Reserved time	Training activity	Aim
Homework	Needs to be executed before the training programme: 120 min	1: Trainees read the guideline 2: Trainees report value of the guideline 3: Trainees send case study 4: Trainees complete knowledge and skills test	1: Trainees start with an equal level of knowledge 2: Trainees are made aware of the value of the guideline in daily practice 3: Training includes case studies which relate to daily practice 4: Trainees realize there is a discrepancy between current behaviour and behaviour according to the guideline
Entry participants	15 min	1: Trainers welcome participants individually, shaking hands 2: Trainees receive a folder with the guideline, summary and programme outline	1: Trainees feel welcome and at ease 2: Trainees are informed about training programme and guideline
Introduction trainers and training programme	10 min	1: Trainers introduce themselves using a PowerPoint presentation 2: Trainers explain their aim of providing a stimulating programme with many learning opportunities 3: Trainers describe the programme	1: Trainees are informed about the role and background of the two trainers (one OP, one IP) 2: Trainees are motivated and energized 3: Trainees are provided with structure
Introduction participants / discuss value of guideline	15 min	Trainees exchange names, their profession and perceived value of the guideline for four minutes with another trainee. After four minutes, trainees switch to another trainee	Trainees become acquainted with other trainees and professions. Discussion of value sets a positive norm concerning the use and value of the guideline and makes trainees realize what value the guideline may have for their work
Coffee break	15 min	NA^a^	Trainees and trainers have a moment to rest and recharge energy levels
Value guideline	30 min	1: Trainers guide plenary discussion of the value of the guideline 2: Trainers guide plenary discussion of their need for knowledge in the training programme	1: Trainees realize what value the guideline may have for their work 2: Training fits trainees’ needs as much as possible
Factors	30 min	1: Trainees work in groups of four (2 OPs/2 IPs) on a case study including influential factors 2: Trainees indicate when to inventory factors on a patient journey in groups of four (2 OPs/2 IPs)	1: Trainees recognize influential factors in a case study 2: Trainees learn when to inventory influential factors
Interventions	30 min	1: Trainees work in groups of four (2 OPs/2 IPs) on a case study 2: Trainees indicate when to use interventions on a patient journey in groups of four (2 OPs/2 IPs)	1: Trainees name and use effective interventions to change negative influential factors 2: Trainees learn that intervention should preferably occur at early stage in the patient journey
Collaboration with employer	40 min	1: Trainees discuss best practices and perform a role play in pairs (1 OP/1 IP) 2: Trainees indicate when collaboration is needed on patient journey (in pairs)	1: Trainees obtain skills to better communicate with the employer 2: Trainees learn when collaboration with the employer is important
Lunch break	60 min	NA^a^	Trainees and trainers have a moment to rest and recharge energy levels
Structure	5 min	Trainers explain the remaining programme	Trainees are provided with structure
Discussion of the cases	30 min	Trainers guide trainee plenary discussion of factors and interventions identified and the reasons for collaboration	Trainees learn from other trainees’ experiences regarding inventory of factors and interventions, and the use of collaboration
Own role of client	60 min	1: Trainees watch a short film 2: Trainers introduce the subject with use of PowerPoint 3: Trainees formulate questions in pairs (either 2 OPs or 2 IPs), which may stimulate the role of individuals with a chronic disease	1: Trainees are introduced to the idea of the client’s own role and obtain knowledge about the value of equal communication between ‘patient’ and doctor 2: Trainees obtain knowledge about the effect on the individual with a chronic disease of being given a role 3: Trainees learn how to stimulate the role of the individual with a chronic disease
Coffee break	20 min	NA^a^	Trainees and trainers have a moment to rest and recharge energy levels
Discussion of patient journey	20 min	Trainers guide plenary discussion regarding the patient journey	Trainees obtain knowledge about when to discuss factors and the early use of an intervention
Individual evaluation of learning objectives	20 min	Trainees write a letter to themselves	Trainees have a reminder of lessons learned in the training programme
Evaluation and closing of training programme	20 min	1: Trainers answer any of the trainees’ remaining questions 2: Trainees evaluate training	1: Trainees are able to share additional questions 2: Trainers acquire insight into trainees’ experiences

^a^NA: Not applicable

### Feasibility

To evaluate feasibility we researched ‘acceptability’, ‘implementation’ and ‘limited efficacy’ as outlined below:

#### Acceptability

To evaluate trainees perspective on the acceptability of the training programme, the OHPs were asked to indicate after the training (T2) to what extent they agreed with four statements on a 10-point visual analogue scale (VAS), with 1 indicating ‘I completely disagree’ and 10 indicating ‘I completely agree’. The statements were: a) ‘Because of the training programme, I am able to use the knowledge and skills provided by the guideline in my own guidance or assessment of people with a chronic disease’, b) ‘The training programme adheres to the daily practice of OHPs in their guidance and assessment of people with a chronic disease’, c) ‘The training programme is relevant to and useful in the guidance and assessment of people with a chronic disease’, d) ‘The training programme contributes to my knowledge and skills concerning the guidance and assessment of people with a chronic disease’. Mean scores and standard deviations were analysed using descriptive statistics (SPSS Statistics 24.0).

#### Implementation

To evaluate trainees perspective on whether the training programme could be implemented on a larger scale, the OHPs were asked to indicate after the training (T2) on a 10-point VAS to what extent the training programme could be implemented in practice, with 1 indicating ‘I completely disagree’ and 10 indicating ‘I completely agree’. In addition, the OHPs were asked to report through open ended questions which barriers to and facilitators they foresaw in implementation of the training programme on a larger scale.

Mean scores and standard deviations on perceived implementation were analysed using descriptive statistics (SPSS Statistics 24.0). Answers to the open-ended questions regarding barriers to and facilitators of implementation were summarized, and similar concepts were grouped together manually by the first researcher (MV). This categorization of similar concepts was checked by the research team (DB, JH, HW, MF).

#### Limited efficacy

To evaluate whether the training programme had an effect, knowledge and skills of OHPs were measured at baseline (T0), after reading the guideline (T1) and directly after completion of the training activities (T2) using knowledge and skills tests. Each test included eight questions, five addressing knowledge and three addressing skills. The latter were addressed by asking the OHPs to apply their knowledge to a case study.

Participants had to give short open-ended answers, which were scored between 0 and 2 points per question. Their performance was evaluated on the basis of the sum of all answers, resulting in a minimum total score of 0 and a maximum total score of 16 points. To achieve consistency and consensus between the researchers, a scoring rubric was used to assess the performance of the participants, which contained all of the correct answers to the questions based on the guideline. This was drafted by the first and second researchers (MV and DB). Both the questionnaire and rubrics were developed by two researchers (MV and DB). Questions and answers included in the tests and rubrics were directly derived from the guideline “Work participation of people with a chronic disease”, to prevent influence of the researchers. The formulated tests and rubrics were checked by the research team (JH, HW, MF).

After the training programme, answers on the questions given by OHPs were scored for correctness by the second researcher (DB) and checked by the first researcher (MV). The total scores per measurement for the entire sample were compared between T0 and T1, and T1 and T2. Since the data were found to have a non-normal distribution, scores were analysed using a non-parametric Friedman test. Post-hoc tests were conducted using Wilcoxon signed rank tests to measure differences between T0 and T1, and T1 and T2 (two-tailed).

## Results

### Participants

A total of 38 participants joined the study, of which 20 worked as OPs, 16 worked as IPs and two worked as both an OP and an IP. An equal number of men (19) and women (19) participated in the study. The average age of the participants was 53 years old (SD: 10), with a range of 26 to 63 years. The OHPs had on average 21 years (SD: 9) work experience, with a range of 0.5 years to 35 years.

### Feasibility

All participants completed the baseline questionnaire (T0) in May 2017. The T1 and T2 questionnaires were also completed by all participants and deployed on the day of the training programme, before the start of the programme (T1) and directly after completion of the training activities (T2). Both training programmes were held in June 2017.

#### Acceptability

Participants reported that the training programme increased their capability to use the guideline (mean: 7, SD: 1). The participants generally found that the training programme adhered to their daily practice (mean: 8, SD: 1) and was relevant to and useful in their guidance and assessment of people with a chronic disease (mean: 8, SD: 1). Finally, the OHPs indicated that the programme contributed to their knowledge and skills related to the guidance and assessment of people with a chronic disease (mean: 8, SD:1).

#### Implementation

The OHPs indicated that the training programme could be implemented on larger scale (mean: 7, SD: 1). However, various barriers to and facilitators of implementation on a large scale were reported. The barriers ‘time’ and ‘money’ were reported to hinder implementation. OHPs also reported that not all managers would give approval for them to undertake the training programme because of organizational constraints.

*Participant: “Managers won’t give permission for employees [occupational physicians or insurance physicians] to take a day off for this [the training programme]”.*

Some OHPs foresaw barriers in relation to the composition of the training programme group. They reported that the size of the group would hinder uptake, or foresaw difficulties with the inclusion of an equal number of OPs and IPs in each training programme group. They also reported that the training programme required active commitment, and that not all OHPs will be motivated to actively participate in the training programme.

Barriers with respect to the content of the guideline were also reported, with some OHPs finding it difficult to read the guideline, or finding the evidence not applicable to every situation. It was also stated that in order for OHPs to use the evidence in practice, more familiarity with it is needed than is provided in a one-day training programme. Finally, several OHPs reported that they foresaw no barriers to the implementation of the training programme on a larger scale.

*Participant: “I don’t see any objections. This [the training programme] is essential for providing a rationale for the recommendations that are given”.*

A frequently reported facilitator was that OHPs were taught the relevance and value of the evidence included in the guideline, as some OHPs had trouble applying the theoretical evidence to their practice. The OHPs also reported that the evidence and training programme provided them with knowledge about and insight into factors and interventions applicable to a broad population. In addition, they reported that a training programme would improve and standardize the guidance and assessment of people with a chronic disease, and that it facilitated the use of knowledge and skills provided in the guideline.

*Participant: “It [the training programme] provides an extra opportunity to gain experience with the guideline. The more often you pick it up and read it, the easier it is to get to grips with.”*

Several OHPs reported that one facilitator of implementation would be the inclusion of both OPs and IPs, as this stimulates trainees to collaborate and learn to work towards one goal, which is optimizing the guidance and assessment of people with a chronic disease. Finally, one OHP suggested that receiving accreditation points would also be a facilitator.

*Participant: “It [the training programme] helps insurance physicians and occupational physicians to speak the same language, which helps improve the collaboration in occupational healthcare and reintegration.”*

#### Limited efficacy

Tests scores on the knowledge and skills tests of the individual participants are displayed in Additional file 1. The non-parametric Friedman test showed a significant improvement in knowledge and skills over time (X^2^ (2) = 53.656, *p* < 0.001), with the median score improving from 6.3 (T0, range: 2–11), to 8.3 (T1, range: 3–13.5), and 12.3 (T2, range: 6–15.5). Post-hoc analysis using the Wilcoxon signed rank test showed a significant improvement between T0 and T1 (p < 0.001), and between T1 and T2 (p < 0.001).

## Discussion

This study examined whether a training programme is a feasible approach to facilitate OHPs’ use of knowledge and skills provided by a guideline. Regarding acceptability, OHPs found that the training programme increased their ability to use the knowledge and skills in daily practice, and they experienced the training programme as useful, relevant and as contributing to their work. The OHPs also indicated that the programme could be implemented on a larger scale, although they foresaw both barriers to and facilitators for implementation on larger scale. The barriers were mainly related to restrictions regarding ‘time’, ‘money’ and the OHPs’ organizational constraints, while the facilitators were related to the added value of the knowledge and skills regarding the guidance and assessment of people with a chronic disease. Also learning to apply the evidence in practice was mentioned as facilitator. Finally, with regard to limited-efficacy, the results showed that the OHPs’ knowledge and skills improved after completing the training programme.

The opinions of the OHPs and their improvement in knowledge and skills highlight the need for a training programme to facilitate the use of knowledge and skills provided by the guideline. These results are congruent with other training programmes facilitating OHPs’ use of knowledge and skills provided by guidelines, including a training programme for IPs [19] and a training programme for OPs [20]. Both programmes have been found to contribute to OHPs’ abilities, with Zwerver et al. [19] reporting improvements in IPs’ attitudes, self-efficacy and intention to apply the knowledge and skills provided by the guideline, while Joosen et al. [20] reported significant improvements in knowledge, self-efficacy and motivation to use the knowledge and skills provided by the guideline.

That the provision of a training programme can be an effective way of facilitating the use of knowledge and skills provided by a guideline has also been confirmed by Michie et al. [12], who indicated that increasing knowledge and skills can also increase capability (‘do OHPs know how to use the knowledge and skills?’) and thereby uptake of OHPs. To increase OHP’s capability, we primarily included training activities (e.g. role play, a case study or discussion of best practices) which reflected daily practice, focusing on learning through personal experience and the ability to discuss issues with peers. Research shows that this approach facilitates the integration of new knowledge and skills with OHPs’ current knowledge base, enhancing the OHPs’ application of knowledge and skills [21, 22].

Although the training programme primarily focused on increasing capability, our results showed that OHPs also found the training programme acceptable, relevant and of value to their work. This may indicate that ‘motivation’ (‘do OHPs believe the knowledge and skills benefit them in their guidance and do they want and plan to use the knowledge and skills?’) is also positively influenced by the programme. As the programme was developed in collaboration with OHPs to ensure that it matched their needs and preferences [16], this may have positively influenced OHPs’ motivation.

With respect to implementation of the training programme on a larger scale, OHPs also reported various barriers and facilitators. These were in line with findings of previous studies, which showed that OHPs primarily reported barriers related to time, money and collaboration with others [10, 11]. Michie et al. [12] includes barriers and facilitators under ‘opportunity’ (‘do OHPs have access to the knowledge and skills and are they supported to use them?’), one of the three conditions that are considered to facilitate uptake. Further implementation should therefore address the barriers and facilitators, as they can largely influence the uptake of the knowledge and skills provided by the guideline on a large scale [16].

A strength of this study is that the training programme included both OPs and IPs. This was done because one of the learning objectives focused on improvement of collaboration between OPs and IPs in their support of people with a chronic disease participating in work. The inclusion of both professions in a training programme had not previously been done, but was perceived as highly beneficial according to our trainees. The OHPs reported this to be a facilitator of the implementation of the training programme, because it supported collaboration and provided the OHPs with the opportunity to learn from each other’s perspectives.

Another strength is that we developed a training programme in collaboration with OHPs, in which we attempted to follow the principles of constructive alignment. By including OHPs in the development of the programme, we aimed to best match the training content and method to the needs of the OHPs, which has proved to positively influence adherence [20, 23]. Previous studies have reported that following the principles of constructive alignment facilitates the integration of knowledge and skills [21, 22]. By doing so, we endeavoured to develop a constructive programme facilitating the use of knowledge and skills by OHPs in daily practice.

A limitation of this study is that we used a one-group pre-post design to measure the increase in knowledge and skills. We decided to not include a control group, as an important learning objective of this training was to stimulate collaboration of OPs and IPs. As OHPs experience a high work load and work in different settings, we decided that a pre-post design approach would serve both the participants, as would provide us with an answer if there is an increase in knowledge and skills. Although we cannot strictly rule out the influence of extraneous variables, we obtained our aim which was to yield trends in the predicted direction for better outcome as per Bowen [17], which is additionally confirmed by OHPs in their perspective on feasibility of the training programme. Future research however should include a control group to exclude the influence of other variables to measure the increase of knowledge and skills.

In addition, the method used to measure knowledge and skills has its limitations, as the training programme and questions were not fully congruent with each other. The taxonomy developed by Bloom et al. [24] classifies different levels of learning, ranging from ‘remembering information’ to the highest level of ‘creating new information’, with the individual being able to produce new information [24]. The training programme primarily focused on applying knowledge, one of the higher levels of learning, whereas the questions used to measure knowledge mainly focused on remembering, the lowest level of learning [24]. Although we attempted to include questions focusing on a higher level of learning by including questions related to skills based on a case study, we were not able to fully match the questions with the programme. We chose this method, as other approaches were not feasible in the chosen setting and time frame of the training programme.

With regard to generalisability, there is a chance that the sample included more intrinsically motivated OHPs since they participated voluntary. However, as trainees received accreditation points (i.e. physicians need to acquire a certain number of accreditation points per year to obtain their registration as a physician) for participation, it is highly likely that many participants joined the training to acquire accreditation points. This means that the sample is likely to be a reflection of the entire population, including both physicians who are intrinsically motivated versus physicians who are primarily motivated by receiving accreditation points.

Future research on implementation and evaluation of the training can expand insight by using a control group or by additional observation of OHPs, allowing us to explore the level of appliance and integration of knowledge and skills by OHPs in daily practice [25]. In addition, the training programme was developed as a one-day programme to make it more feasible for OHPs to attend and to fit with their daily practice. As research shows that recall and use of knowledge and skills can diminish over time [26], it might be worth considering the addition of follow-up meetings aimed to increase the recall of OHPs. Further research might therefore also explore whether a training programme containing multiple sessions or including follow-up meetings is more effective while remaining a feasible approach for OHPs.

## Conclusion

This study evaluated the feasibility of a training programme to facilitate OHPs’ use of knowledge and skills provided by a guideline. The results of the study showed that OHPs considered the training programme to be feasible, and that the OHPs’ knowledge and skills increased after completing the training programme. Thus, the programme can serve as an approach to facilitate OHPs’ use of knowledge and skills provided by a guideline.

## Additional file

Additional file 1: Includes data titled ‘individual scores of participants on T0, T1 and T2 (limited efficacy)’. To research if the training showed limited efficacy, we measured the increase in knowledge and skills of the participants, by administering knowledge and skills tests at baseline (T0), before the training (T1) and after the training (T2). The supplementary files shows the scores of all participants on each test (T0, T1 and T2). (DOCX 14 kb)

## Abbreviations

IPs: Insurance physicians
M: Mean
NA: Not applicable
OHPs: Occupational health professionals
OPs: Occupational physicians
SD: Standard deviation
VAS: Visual analogue scale
