# Supplementary material for: A training programme facilitating guideline use of occupational health professionals: a feasibility study
Source: BMC Med Educ. 2018 Oct 3;18:226. doi: 10.1186/s12909-018-1223-1 (PMC6169000; doi:10.1186/s12909-018-1223-1)
Supplement: Supplementary file 1 — Includes data titled ‘individual scores of participants on T0, T1 and T2 (limited efficacy)’. To research if the training showed limited efficacy, we measured the increase in knowledge and skills of the participants, by administering knowledge and skills tests at baseline (T0), before the training (T1) and after the training (T2). The supplementary files shows the scores of all participants on each test (T0, T1 and T2). (DOCX 14 kb) [file 12909_2018_1223_MOESM1_ESM.docx]

Appendix A: Individual scores of participants on T0, T1 and T2 (limited efficacy)

| Respondent | Total score T0 | Total score T1 | Total score T2 |
| --- | --- | --- | --- |
| 1 | 2 | 7 | 11,5 |
| 2 | 6 | 10 | 14 |
| 3 | 6 | 7,5 | 13 |
| 4 | 3,5 | 7 | 6 |
| 5 | 9 | 6,5 | 10 |
| 6 | 8 | 5 | 14 |
| 7 | 5,5 | 8 | 15 |
| 8 | 2,5 | 6 | 10 |
| 9 | 7,5 | 9 | 10 |
| 10 | 6,5 | 5,5 | 9,5 |
| 11 | 7 | 7 | 11,5 |
| 12 | 4 | 7 | 10 |
| 13 | 6,5 | 10,5 | 12 |
| 14 | 2 | 3 | 10 |
| 15 | 7,5 | 13,5 | 13 |
| 16 | 4 | 6,5 | 13,5 |
| 17 | 6 | 9 | 10 |
| 18 | 4,5 | 7,5 | 9,5 |
| 19 | 5,5 | 8,5 | 13 |
| 20 | 8 | 13 | 13,5 |
| 21 | 5,5 | 7 | 12 |
| 22 | 7 | 12 | 13,5 |
| 23 | 11 | 7 | 15 |
| 24 | 8,5 | 10,5 | 9,5 |
| 25 | 7,5 | 7 | 10,5 |
| 26 | 8,5 | 10,5 | 10 |
| 27 | 7 | 10,5 | 12,5 |
| 28 | 7,5 | 10,5 | 14,5 |
| 29 | 5 | 6 | 10,5 |
| 30 | 8 | 10,5 | 15 |
| 31 | 3 | 10 | 14 |
| 32 | 6 | 3 | 7 |
| 33 | 6 | 6,5 | 14,5 |
| 34 | 9 | 8,5 | 13 |
| 35 | 3 | 12 | 15,5 |
| 36 | 5,5 | 11 | 10,5 |
| 37 | 8,5 | 12 | 13 |
| 38 | 7 | 9 | 15 |
